# Address

**Published:** 1866-01

**Authors:** H. F. Bishop


### ADDRESS.

[Read before the Mass. Dental Association at Boston.

H. F. BISHOP, D.D.S.

NEARLY three hundred years ago Cervantes makes Don Quixotte say: "a diamond is not so precious as a tooth." There have been wonderful developments in dental science since Sancho Panza was called to officiate as a dentist to his noble master Don Quixotte. Rude aud unskilled were the fingers of that age in comparison with those now required to perform the varied duties of a dentist; but now, as *then*, a tooth is more precious than a diamond.

*Dental science of to-day—or dental education*—is the theme of this paper.

No student, of however high qualification, can enter upon the task of acquiring professional excellence in this noble calling without engaging, at once, in a career of great labor, demanding the utmost perseverance through a long life.

The requirements of a dental education, which shall meet the demands of the present age, are manifold. Dentistry, both as a specialty of surgery or operative dentistry, and as a mechanical art—the two covering the least possible ground of attainment necessary for admission into the profession, will herein be considered. As a specialty of surgery, how extensive and diversified is the field of labor, which keeps the student forever passing over new ground ; never the same operation under the same pathological conditions—never, or almost never, the same normal or abnormal condition of things existing twice, revealing what treatment shall govern us.

Dentists are not fully qualified for their profession unless they have devoted much attention to the general subject of medicine and surgery ; I would that all should graduate hereafter, but, if not, they cannot excuse themselves from a thorough knowledge of anatomy, physiological chemistry, pathology and therapeutics.

For is not a dental student expected to understand the anatomy of the human body thoroughly ? to be familiar with the bones and the muscles—to understand their mechanism, their relative position and functions ? see clearly their connection between those life-giving organs of respiration, circulation and digestion. Nor may he be ignorant of the functions or rules of other organic or inorganic forces ; secretion, absorption and perspiration are all to be traced out in the circulation of their fluids in supplying the needed life to the system. These must all be understood as well as the exhaustion or waste of such fluids when devitalized.

Then into how many hidden paths of microscopic research must he work his way and investigate the capillary and tubulated mechanism of a tooth from its original gum or sac to its final perfection.

He must follow the development of the membrane through its ingenious method of clothing itself for perfection with those harder substances of bone, enamel and cementum. All this and much more is requisite to obtain a starting point to the great field lying before the student of dentistry. These last are not specialties in which the student of medicine or surgery keeps pace with the dental surgery—while we must have his generalities, he excuses himself from our specialties. While we cannot dispense with our colleges and dental educators for ourselves, we should be invited to fill a chair in their colleges, for the purpose of teaching a branch of their science almost wholly neglected. I desire to look upon the bright side of this matter—and perhaps some would say that I over-estimate both my profession and those engaged in it; but I consider it a duty we owe to ourselves to keep up a high standard of dental qualification. It is my great joy and satisfaction that I speak to a highly intelligent, and I trust, educated body. I hope the day is past for those to come into our ranks who have had no advantages of early education; and I believe that such as we have had are already far on the high road to respectable attainments by self culture and indomitable perseverance. How many still among us who have been in practice ten, or even twenty years, will hesitate to take a course of lectures in our dental colleges? If I am allowed to speak from my own experience, I can truly say that after ten years of practice, I found it exceedingly profitable to attend college, and now, after ten years more of practice, since graduation, I can hardly see how I can afford to stay away from those golden opportunities of instruction which these institutions offer.

Now, dental education, requiring such diversity of talent as it does, may be subserved, and both operators and students be benefited by a survey of those engaged in practice; especially those who, in one department or another, have reached some professional eminence—who have some superior excellence in their own method—who give the best results in particular departments, in which they have made some discovery or achievement valuable hereafter to all. The highest skill and ability of the most eminent and successful in our profession should be our patterns till we may obtain a more excellent way of our own: and while we feel stimulated by what has been done, let us *study*, read, *think* and work for those hidden truths yet to be revealed. While few as yet have attained very satisfactory results of the profession, yet no one need despair of having some special thunder of his own which should be his mark of distinction—which should give him some respect with his professional brethren. Either he should have invented or discovered some useful aid to our calling—have given us some easy way to accomplish some hitherto difficult operation; or he may have the credit of being particularly skilled in the case of the natural teeth, or be able to render the best possible substitutes for the loss of them. Let him give us a treatise on dietetic influences on the teeth—let him classify the evils or benefits suffered by or secured to children of certain ages—let him show us the best possible results in the preservation of the deciduous teeth and their wonderful relation to the second set—or he may commence still earlier and show what beneficial results may be secured by parents for their offspring even before birth, and draw correct conclusions from congenital life to our help.

The greatest wisdom and skill are necessary in the case of teeth between the ages of eight and twenty, and he who is wise in the diagnosis of the requirements of a patient at this important age — seing not only what is presented to the eye to-day, but foreseeing the future, and taking measures accordingly to secure a healthy denture, is not destitute of a high order of talent for the practice of dentistry.

The present state of dental education suggests the practicability of a sub-division of labor, and renders it almost necessary that there should be specialists for the highest attainments in our most difficult departments. Not every one could be expected to undertake successfully, cleft palates, fractured jaws and diseased antrums; and even orthodontia, or the treatment of obstinate irregularities and the filling of difficult teeth require a specialist almost as much as the construction of artificial velums for cleft palates.

Mechanical dentistry, too, is a field affording abundant room for skill in supplying dental substitutes not only, but leads one at once into a great variety of collateral departments of mechanism where the inventive faculty has full scope.

There is no profession or calling which demands greater diversity of talent or more familiarity with mechanical art and science than dentistry. The student who contemplates the mastery of this profession should never think of giving less than years of time and devotion for preparation — both with able preceptors in private practice, and by attending two courses of lectures, or an equivalent in our colleges. There is no shorter or easier method to final success than thorough preparation; nor need he feel that he has to wait for age and grey hairs, with long and valuable experience before he can be in successful practice. Experience is of great value, and not to be underrated, but still the great advantages of education which our colleges afford, together with all the helps a student can command in these days, should qualify one for great superiority in work as compared with years in the past.

I trust the day is past for a quack in dentistry to stand in any desirable position, or to make it long profitable in a pecuniary sense. One great education for all of us is a thorough digestion of our dental periodicals ; they should be held indispensable and although now of quite high order might be still more and more improved by contributions from the daily laborers and gleaners in the field. Other great educators, also, are the dental societies which are springing up all over the country ; he who abstains entirely from these useful discussions by associated bodies holds no enviable position. He is not willing to benefit either himself, his profession or mankind generally. It is a lamentable fact that in too many cities dentistry suffers from want of associated labor, and the co-operation of those engaged in it. Can it possibly be a sufficient excuse for men who have long labored honorably and successfully in the profession, to stand aloof from the meetings of societies and conventions, instituted for the elevation and development of the profession, because, perhaps, there may be men in these associations with whom they disdain to fraternize ? Why do they not shake off this Jewish robe of seclusion, and come forth among the gentile host, which truly has the ark of the profession in charge to hand down to posterity, and lend their dignity and influence to make these societies, if not what they like, such as they think they *should* be, in character and standing ?

In concluding this paper, gentlemen, allow me to ask, what shall be our governing rules in regard to taking students ? Will not every man in the profession pledge himself, hereafter, to exact a long pupilage from every student, and offer every inducement and encouragement to students to graduate before commencing practice.

Henceforth, let our motto be excelsior, — remembering, that success, in the highest acceptation of the word, comes only through strict honesty. Let us be honest to ourselves, — honest to the profession — honest to our patients — honest to facts and truths, and those great facts and truths will soon crown our hopes with success, equal to our highest ambition, and place our profession where it shall be, on a footing with the liberal professions of the country.

